# Cardiovascular Diseases and Type 2 Diabetes: 2nd Edition

**DOI:** 10.3390/medicina62102034

**Published:** 2026-10-21

**Authors:** Cristina Tudoran

**Affiliations:** 1 Centre of Molecular Research in Nephrology and Vascular Disease, “Victor Babes” University of Medicine and Pharmacy Timisoara, Eftimie Murgu Square No. 2, 300041 Timisoara, Romania; tudoran.cristina@umft.ro; 2 Department VII, Internal Medicine II, Discipline of Cardiology, “Victor Babes” University of Medicine and Pharmacy Timisoara, Eftimie Murgu Square No. 2, 300041 Timisoara, Romania; 3 County Emergency Hospital “Pius Brinzeu”, L. Rebreanu, Nr. 156, 300723 Timisoara, Romania

Diabetes mellitus (DM) is associated with multiple comorbidities, the most important being cardiovascular (CV) diseases (CVDs) and especially coronary artery disease (CAD), peripheral artery disease (PAD), stroke, and chronic kidney disease (CKD) [1,2,3,4]. Although the well-known relationship between DM and CVD is bidirectional, it is more complex than initially considered. On the one hand, type 2 DM (T2DM) represents an independent risk factor for the development of atherosclerosis, CAD, PAD, systemic hypertension (SH), and chronic heart failure (CHF), intervening through multiple pathways in the pathophysiology of CVD. On the other hand, the coexistence of CVD represents a challenge for the management of DM, increasing the risk for other complications [5,6,7].

The first part of this Special Issue details the interconnection between microvascular alterations in diabetic patients. In particular, T2DM favours the development of endothelial dysfunction (ED), which is responsible for the occurrence of subsequent CV complications or may aggravate pre-existing pathologies [8]. A study based on data from the European Health Interview Surveys in Hungary, carried out on 15,874 individuals, demonstrated that T2DM was significantly associated with higher prevalence rates of hypertension (by 23.4%), arrhythmias (by 3.85%), heart attack (by 3.42%), and CAD (by 6.92%) after adjusting for potential confounders (contribution 1).

Vitamin D deficiency has been recognised as a potential contributing factor to vascular disease. Even in patients with T2DM without a clinically evident diagnosis of CVD, marked elevations in parameters characterising arterial stiffness (AS) (intima–media thickness and aortic pulse wave velocity), decreased serum levels of 25-hydroxyvitamin D3 (25(OH)D3), and a diminished left ventricular (LV) global longitudinal strain (LVGLS) in comparison to healthy subjects were shown [9]. Furthermore, data suggest that an elevated AS correlates with detrimental remodelling of the LV even in individuals with preserved ejection fraction, thereby strengthening the impact of vascular–ventricular coupling on the development of diabetic cardiomyopathy (contribution 2). Another factor that could contribute to the increased AS in patients with T2DM is indoxyl sulfate (IS), a gut microbiota-derived metabolite of tryptophan, which could be implicated in vascular dysfunction through oxidative stress and inflammation [10]. Serum IS concentrations are independently associated with aortic stiffness (AoS) in patients with T2DM, underscoring the role of IS in the development of vascular complications in T2DM. The results emphasise the need to consider nontraditional risk factors beyond standard CV assessments and support the potential clinical utility of IS measurement for improved risk stratification (contribution 3) [10].

The second part of the Special Issue analyses macrovascular alteration in patients with T2DM. In their study, Boieru et al. demonstrated that the burden of CAD (as indicated by the Synergy Between Percutaneous Coronary Intervention with Taxus and Cardiac Surgery (SYNTAX) score) is more pronounced in patients with T2DM, especially in populations with severe CAD, such as individuals scheduled for coronary artery bypass grafting (CABG) [11] (contribution 4). Although ED, assessed through flow-mediated vasodilatation, and circulating 5-hydroxytryptamine (5-HT) levels are more pronounced in diabetic patients, the circulating levels of cytosolic copper/zinc superoxide dismutase (SOD-1) and lectin-like oxidised low-density lipoprotein receptor-1 (LOX-1), which reflect oxidative stress, are not influenced by T2DM in patients with severe CAD (contribution 4) [12].

Diabetic retinopathy (DR) is a leading cause of blindness in patients with T2DM. CV risk factors, such as SH, obesity, and dyslipidaemia, may play a crucial role in the development and progression of DR, though the evidence remains inconclusive [13]. In their retrospective cross-sectional study of 377 diabetic patients, Rosu et al. analysed CV risk profiles in patients with DR and T2DM and tried to develop a predictive model for the progression of DR (contribution 5). Among the tested machine-learning models, XGBoost demonstrated the best performance with an ROC-AUC of 0.72, identifying systolic and diastolic blood pressure, high-density lipoprotein (HDL) cholesterol, and body mass index (BMI) as the most important risk factors (contribution 5).

The third part of this collection analyses several particularities of patients with DM and CVD. It studies the impact of DM and/or sequelae of myocardial infarction (MI) on the dysfunction of the autonomic nervous system (ANS) [14]. Various degrees of ANS dysfunction (predominantly parasympathetic impairment) were observed across different categories of patients: diabetic patients following MI, patients with MI without DM, and diabetic patients without MI. These abnormalities were most pronounced in diabetic patients recovering from MI, suggesting a cumulative detrimental effect of DM and MI on autonomic regulation [14]. In this group of individuals, non-ST-elevation MI and abnormal Valsalva manoeuvre responses, with impaired cardiovagal function and baroreflex sensitivity, indicated increased long-term mortality (contribution 6).

Beyond the well-recognised LV involvement in diabetic cardiomyopathy, emerging data indicate that subclinical right ventricular (RV) dysfunction may also be present in patients with T2DM and CVD. These data led researchers to investigate more thoroughly the presence of subclinical RV dysfunction by using a comprehensive transthoracic echocardiographic evaluation encompassing conventional parameters (tricuspid annular plane systolic excursion (TAPSE), tricuspid annular systolic velocity (TV S’), right ventricular fractional area change (RVFAC)) and deformation imaging using speckle-tracking (RV global longitudinal strain (RVGLS), RV free-wall longitudinal strain (RVFWS)) [15,16]. Subclinical RV systolic dysfunction may not be detected by traditional echocardiographic indices (contribution 7). Speckle-tracking echocardiography identified these early changes, showing reduced RVGLS and RVFWS values, with RVGLS providing the strongest diagnostic performance (AUC 0.84) and a practical threshold around −17% (sensitivity ~86%, specificity ~81%) (contribution 7).

The fourth part of this Special Issue focuses on the impact of COVID-19 on CV pathology [17] and, especially, on the particularities encountered in diabetic patients. Viral infections have often complicated glycaemic control in diabetic patients [18]. During the COVID-19 pandemic, dysglycaemia was established as a major determinant of adverse outcomes, yet the separate contributions of poor glycaemic control and glycaemic variability (GV) remained incompletely understood [18,19]. Pah et al. conducted a systematic review and meta-analysis of observational cohort studies and determined that both chronic hyperglycaemia and elevated GV are associated with increased risk of severe COVID-19 outcomes (contribution 8). GV appears to be a consistent, low-heterogeneity prognostic marker of mortality, being separately associated with a higher death risk in hospitalised COVID-19 patients, highlighting its potential as a dynamic metabolic biomarker. These observational findings support the necessity of structured glucose surveillance, including continuous glucose monitoring in hospitalised COVID-19 patients and, especially, in those treated in intensive care units. GV should therefore be interpreted primarily as a high-risk prognostic marker rather than definitive evidence of a causal treatment target (contribution 9).

Another study demonstrated that DM was associated with a higher burden of CV comorbidities and impaired renal function at admission in hospitalised adults with SARS-CoV-2 infection. Their findings suggest that DM could be seen as a marker of clustered cardiometabolic and renal vulnerability, reinforcing the need to focus on objective severity and organ dysfunction markers while still recognising diabetic patients as a high-risk group requiring close monitoring and optimised care (contribution 9).

The convergence of continuous glucose monitoring (CGM) technology, fasting biology, and longevity pharmacology represents a promising future research direction for preventive cardiology in apparently healthy individuals. In their review, Vacarescu et al. synthesise evidence connecting CGM-guided metabolic self-knowledge with preventive cardiology and longevity medicine (contribution 10). Evidence shows that CGM provides not merely a diagnostic window, but an actionable behavioural feedback mechanism that, when combined with structured fasting protocols and longevity-oriented pharmacology (low-dose metformin and acarbose), constitutes a comprehensive preventive strategy accessible to apparently healthy individuals (contribution 10).

Future studies should concentrate on multidisciplinary approaches and targeted interventions to reduce CV risk in diabetic populations. Consequently, healthcare systems should adopt integrated care models targeting metabolic and CV parameters, such as autonomic testing, new parameters characterising AS, etc., to allow for a faster and more efficient CV risk assessment in patients with DM (contributions 1, 2, 4–6). Integrating modern parameters for RV dysfunction (especially RVGLS) into routine echocardiographic evaluation could enhance early detection, support timely intervention, and ultimately improve outcomes in patients with T2DM and CVD (contribution 7) [15,20]. The results presented in this Special Issue could guide the development of biomarkers and new therapies to reduce CV risk in this high-risk group (contribution 3).

Targeting vitamin D3 deficiency may be relevant not only for metabolic health but also as an early intervention to reduce CV risk. Further prospective, large-scale studies are needed to determine whether the correction of vitamin D deficiencies could improve metabolic health and reduce AS, ameliorating CV outcomes in this high-risk population (contribution 2). Large double-blinded randomised trials should focus on the importance of 5-HT in patients with CAD and T2DM (contribution 4).

Although the COVID-19 pandemic has passed, the SARS-CoV-2 virus is still present worldwide, and COVID-19 has the potential to cause severe complications in high-risk patients (contributions 8, 9). This is why COVID-19 research should continue, especially among patients with T2DM. Patients with CVD and DM presenting with acute respiratory insufficiency should also be tested for the SARS-CoV-2 virus.

## List of Contributions

1. Pataki, J.; Szőllősi, G.J. Impact of Diabetes on Excessive Cardiovascular Risk: Matched Analysis Based on the European Health Interview Survey. *Medicina* **2024**, *60*, 1928. https://doi.org/10.3390/medicina60121928.
2. Craciun, L.M.; Buleu, F.; Iurciuc, S.; Popa, D.I.; Pop, G.N.; Goanta, F.; Goje, G.-I.; Pah, A.M.; Badalica-Petrescu, M.; Bodea, O.; et al. Arterial Stiffness and Early Cardiac Dysfunction in Type 2 Diabetes Mellitus: A Potential Role for 25 OH Vitamin D3 Deficiency. *Medicina* **2025**, *61*, 1349. https://doi.org/10.3390/medicina61081349.
3. Su, I.-M.; Teng, Y.-Y.; Li, J.-C.; Liu, C.-H.; Wu, D.-A.; Hsu, B.-G. Serum Indoxyl Sulfate as a Potential Biomarker of Aortic Stiffness in Persons with Type 2 Diabetes Mellitus. *Medicina* **2025**, *61*, 1607. https://doi.org/10.3390/medicina61091607.
4. Boieriu, A.M.; Luca, C.D.; Neculoiu, C.D.; Bisoc, A.; Țînț, D. Endothelial Dysfunction and Oxidative Stress in Patients with Severe Coronary Artery Disease: Does Diabetes Play a Contributing Role? *Medicina* **2025**, *61*, 135. https://doi.org/10.3390/medicina61010135.
5. Roşu, C.D.; Bratu, M.L.; Stoicescu, E.R.; Iacob, R.; Hațegan, O.A.; Ghenciu, L.A.; Bolintineanu, S.L. Cardiovascular Risk Factors as Independent Predictors of Diabetic Retinopathy in Type II Diabetes Mellitus: The Development of a Predictive Model. *Medicina* **2024**, *60*, 1617. https://doi.org/10.3390/medicina60101617.
6. Marković, N.; Petrović, M.; Žugić, V.; Bulatović, S.; Bojić, M.; Milovanović, B. Autonomic Nervous System Dysfunction in Diabetic Patients After Myocardial Infarction: Prognostic Role of the Valsalva Maneuver. *Medicina* **2026**, *62*, 96. https://doi.org/10.3390/medicina62010096.
7. Benchea, L.-C.; Anghel, L.; Dubei, N.; Zanfirescu, R.-L.; Bîrgoan, G.-S.; Sascău, R.A.; Stătescu, C. Unmasking Subclinical Right Ventricular Dysfunction in Type 2 Diabetes Mellitus: A Speckle-Tracking Echocardiographic Study. *Medicina* **2025**, *61*, 1516. https://doi.org/10.3390/medicina61091516.
8. Pah, A.-M.; Gavrilescu, D.-M.; Mateescu, D.-M.; Cotet, I.-G.; Craciun, M.-L.; Florescu, E.; Crisan, S.; Avram, A. Association of Chronic Hyperglycemia and Glycemic Variability with Mortality in COVID-19: Meta-Analysis of Cohort Studies. *Medicina* **2026**, *62*, 310. https://doi.org/10.3390/medicina62020310.
9. Cotet, I.-G.; Toma, A.-O.; Mateescu, D.-M.; Ilie, A.-C.; Ursoniu, S.; Surducan, D.A.; Gridan, C.; Iurciuc, S.; Gavrilescu, D.-M.; Tudoran, C. Diabetes Mellitus and In-Hospital Outcomes in Hospitalized COVID-19 Patients: A Single-Center Eastern European Cohort Study (2020–2024). *Medicina* **2026**, *62*, 410. https://doi.org/10.3390/medicina62020410.
10. Văcărescu, C.; Cozma, D. Beyond Diabetes: Continuous Glucose Monitoring as a Candidate Precision Tool for Cardiovascular Prevention and Healthy Longevity—A Hypothesis-Generating Narrative Review. *Medicina* **2026**, *62*, 1513. https://doi.org/10.3390/medicina62081513.
